# Newly Emerging Nanotechnologies of Innovative Devices for Radioisotope Batteries

**DOI:** 10.3390/nano16090511

**Published:** 2026-04-23

**Authors:** Qiang Huang, Shaopeng Qin, Runmeng Huang, Xue Yu, Junfeng Zhang, Guohui Liu, Haixu Zhang, Ming Liu, Sijie Li, Xue Li, Xin Li

**Affiliations:** Department of Isotope, China Institute of Atomic Energy, Beijing 102413, China

**Keywords:** radioisotope batteries, nanotechnology, energy conversion, thermoelectric materials, wide-bandgap semiconductors

## Abstract

Nanotechnology has emerged as a key driver in radioisotope batteries, which offer unique advantages for long-term, maintenance-free energy supply in deep space exploration, medical implants, and nuclear waste utilization. This review summarizes recent progress in applying nanomaterials and nanostructures to overcome the limitations of nuclear batteries, including low energy conversion efficiency and poor stability. The main content focuses on the three primary conversion mechanisms of thermoelectric, radio-voltaic, and radio-photovoltaic batteries, discussing high-performance thermoelectric nanomaterials such as SiGe alloys, wide-bandgap semiconductors including diamond and SiC for enhanced carrier collection, and nanoscale radionuclide ources to mitigate self-absorption losses. This review further elaborates on how nanostructure regulation and interface engineering have significantly improved carrier collection efficiency and device stability. These advances have enabled notable civilian applications, such as the BV100 and “Zhulong No.1” nuclear batteries. Despite this progress, challenges remain in ensuring long-term material stability under extreme environments, maintaining performance consistency during macroscopic device integration, and addressing the high fabrication costs. The review concludes by outlining future research directions, including the development of novel nanomaterial systems, innovative nanostructure designs, scalable manufacturing processes, and enhanced device stability and safety, to further advance next-generation radioisotope batteries.

## 1. Introduction

**Figure 1:**
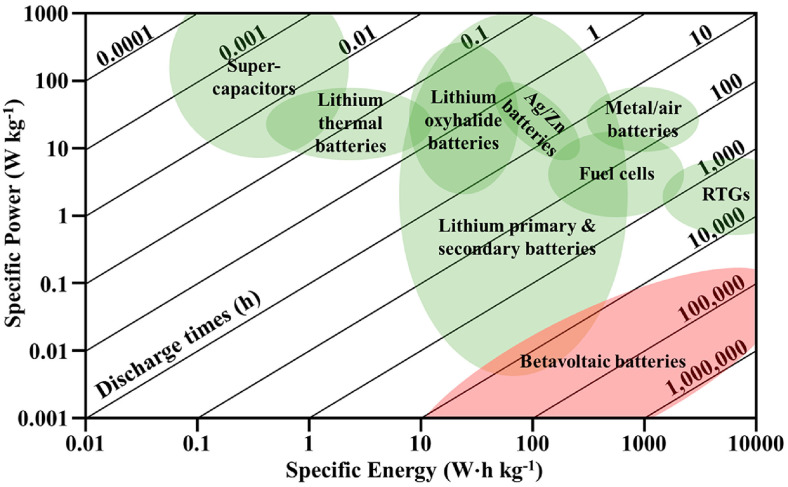
Ragone plot of various batteries [4].

Energy has played a vital role in the development of science and technology [1]. Nuclear energy is a clean and promising energy carrier that is expected to speed up scientific progress in the future. A radioisotope cell, also called an atomic battery or nuclear battery, is a device that converts nuclear energy to electric energy by harnessing high-energy particles emitted from the spontaneous decay of radioactive materials (such as Pu-238, Sr-90, Ni-63, and C-14) [2,3,4,5]. The lifetime of the radioisotope battery is closely related to the half-life of the radionuclide, which can range from several decades (e.g., Ni-63 has a half-life of 100 years) to thousands of years (e.g., C-14 has a half-life of 5730 years), far exceeding that of conventional energy storage devices such as lithium-ion batteries and supercapacitors [6,7,8]. Meanwhile, the spontaneous decay of radionuclides is a continuous process and is free from the influence of the environment. These inherent characteristics make radioisotope batteries irreplaceable in scenarios requiring a long-term stable energy supply without maintenance, especially in extreme environments where regular maintenance is impossible (Figure 1).

Along with the rapid development of aerospace exploration, microelectronics, medical devices, and nuclear waste resource utilization, the demand for high-performance, miniaturized, and multi-scenario-adaptable radioisotope batteries has continued to grow exponentially [8,9]. In the field of aerospace, radioisotope thermoelectric generators (RTGs) have become the core energy system for deep space exploration missions (such as lunar and Martian exploration), as they can operate stably in extreme environments such as ultra-low-temperature (about −180 °C during the lunar night), high radiation (10^5^–10^7^ Gy), and vacuum environments where solar batteries and chemical batteries have difficulty functioning [10,11]. For instance, the Chang’E-3 and -4 lunar rovers adopted radioisotope batteries with a power of 0.1–2 W to ensure normal operation during the lunar night, which verified the reliability of radioisotope batteries in deep space exploration [12,13]. In the medical and microelectronics field, miniaturized radioisotope batteries have shown broad application prospects in biomedical implants (such as cardiac pacemakers, which require 10–50 μW of continuous power supply for 10–20 years), Internet of Things (IoT) sensors (especially for remote areas and harsh environments), and micro-robots, where a long-term, low-power, and maintenance-free energy supply is required [14,15]. Based on the increasing demand for such batteries, various research groups have conducted extensive research and announced advances, such as the BV100 miniature nuclear battery from Beijing Betavolt [15]. Additionally, radioisotope batteries also play a crucial role in the resource utilization of nuclear waste. The decay energy of hazardous nuclear waste can be converted to electric energy through radioisotope batteries, thus reducing the long-term radioactive hazards [7].

Despite the advantages mentioned above, radioisotope batteries also face severe technical bottlenecks, including a relatively low radionuclide utilization rate and energy conversion efficiency, insufficient performance stability, and difficulties in miniaturization and integration, which restrict their large-scale applications [4,8,11,16]. As for the relatively low radionuclide utilization rate and energy conversion efficiency, the self-absorption effect of radionuclide sources (especially for α particles with short range) is a significant factor, which causes a 70–90% loss of decay energy within the source layer [17]. Meanwhile, the low thermoelectric figure of merit (ZT < 1.0 at high temperature) of traditional thermoelectric materials (such as bulk SiGe alloy) and the low carrier collection efficiency (less than 30%) of planar semiconductor structures further limit the overall energy conversion efficiency [9]. For example, the energy conversion efficiency of traditional silicon-based β-voltaic batteries is usually less than 1%, and the conversion efficiency of actinide-based radioisotope batteries is even lower (0.1–0.5%) due to severe self-absorption [4,18]. The relatively low radionuclide utilization rate and energy conversion efficiency further increase the cost of radioisotope batteries, which makes it difficult to popularize radioisotope batteries in civilian fields [3,8]. The physicochemical properties of radioisotope battery materials suffer from extreme environments (such as high-temperature, high-radiation-dose-rate, and harsh chemical conditions), leading to lattice distortion and interface damage, thus reducing service life and output stability [4,6]. Due to difficulties in miniaturization and integration, traditional radioisotope battery structures are relatively bulky (with volumes usually greater than 1 cm^3^), and it is difficult to balance miniaturization with performance, which restricts their application in microelectronic devices and implantable medical equipment.

With the rapid progress in nanotechnology, the research on radioisotope batteries has grown active again. Nanotechnology, which focuses on the precise construction and manipulation of materials and devices at the atomic/molecular scale (1–100 nm), can significantly optimize the structure and performance of radioisotope battery components (including radionuclide sources, energy conversion materials, and interface layers) by regulating the size, morphology, and interface properties of nanomaterials [1,15]. Compared with bulk materials, nanomaterials exhibit unique physical and chemical properties, such as high specific surface area, quantum size effect, and interface effect, which can effectively enhance the absorption and utilization of decay particles, improve the thermoelectric performance and radiation resistance of energy conversion materials, and realize the miniaturization and integration of devices [19]. In the past decades, researchers have carried out extensive and in-depth research on the application of nanotechnology in radioisotope batteries, achieving a series of breakthroughs in nanomaterial system innovation, nanostructure regulation, and device performance optimization [17,18,20,21,22]. This review summarizes recent progress in applying nanomaterials and nanostructures to overcome the limitations of nuclear batteries, including low energy conversion efficiency and poor stability. The main content focuses on the three primary conversion mechanisms of thermoelectric, radio-voltaic, and radio-photovoltaic batteries, discussing high-performance thermoelectric nanomaterials such as SiGe alloys, wide-bandgap semiconductors including diamond and SiC for enhanced carrier collection, and nanoscale radionuclide sources to mitigate self-absorption losses. This review further elaborates on how nanostructure regulation and interface engineering have significantly improved carrier collection efficiency and device stability. These advances have enabled notable civilian applications, such as the BV100 and “Zhulong No.1” nuclear batteries. Despite this progress, challenges remain in ensuring long-term material stability under extreme environments, maintaining performance consistency during macroscopic device integration, and addressing the high fabrication costs. The review concludes by outlining future research directions, including the development of novel nanomaterial systems, innovative nanostructure designs, scalable manufacturing processes, and enhanced device stability and safety, to further advance next-generation radioisotope batteries.

## 2. Brief Overview of Electricity Conversion in Radioisotope Batteries and Inherent Limitations

A radioisotope battery is typically composed of three essential components: the radioisotope source(s), the radiation transport medium, and the energy conversion transducer(s) [4,5,8]. Based on the decay energy utilizing pathway, three main radioisotope battery categories are commonly recognized: thermoelectric conversion radioisotope batteries, radio-voltaic batteries, and radio-photovoltaic batteries [3,4,8,23]. Each pathway presents unique operational principles, advantages, and fundamental limitations that govern their applicability across different technological domains. This section briefly describes the basic mechanisms of these three types of radioisotope batteries and analyzes the key bottlenecks that restrict performance improvement, laying a foundation for the subsequent discussion of how nanomaterials and nanostructures resolve these issues.

### 2.1. Thermoelectric Conversion Radioisotope Batteries

**Figure 2:**
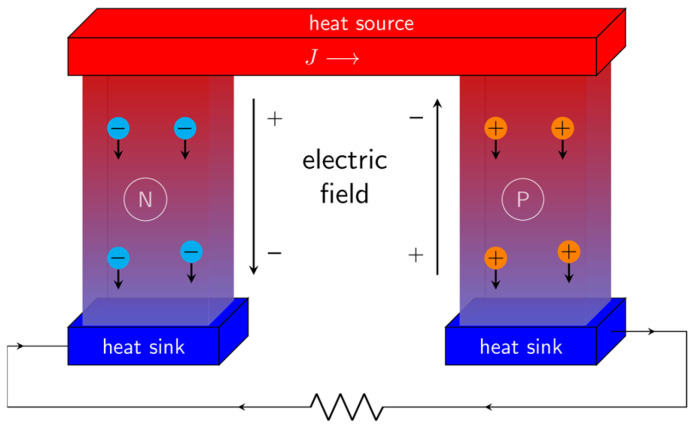
General mechanism of Seebeck effect.

Thermoelectric conversion radioisotope batteries, exemplified by the RTG, are the most mature and widely deployed radioisotope batteries, having powered numerous deep space missions including the Voyager, Cassini, and New Horizons spacecraft [24]. The general conversion mechanism is based on the Seebeck effect (Figure 2) [25], which involves three steps: first, a radioactive isotope source (such as Pu-238 source) releases heat through spontaneous decay; second, this heat raises the temperature of a “hot side” in direct contact with the source, while a “cold side” is maintained at a lower temperature via passive radiative cooling, establishing a stable temperature gradient; third, thermoelectric modules composed of n-type and p-type semiconductors convert this temperature difference into electricity–electrons drift through n-type materials and holes through p-type materials, generating a net voltage and sustained current in an external circuit. The efficiency of this process is governed by the thermoelectric material’s dimensionless figure of merit, *ZT* = (*S*^2^*σT*)/*κ*, where *S* is the Seebeck coefficient, *σ* is the electrical conductivity, *T* is the absolute temperature, and *κ* is the thermal conductivity. Hence, such batteries have the advantages of stable and reliable operation, a robust structure, and no requirement for a starting device, while simultaneously being constrained by the properties of thermoelectric materials.

For decades, the benchmark materials for RTGs have been bulk SiGe alloys and PbTe/TAGS systems, which offer adequate stability at elevated operating temperatures but suffer from limited *ZT* near or below 1.0. For instance, n-type SiGe alloys achieve a *ZT* of approximately 1.0 at 1000 K, a performance metric that has remained largely unchanged for decades. The intrinsic limitation lies in the strong coupling of *S*, *σ*, and *κ_e_*: improving one parameter often leads to the degradation of another. For example, increasing the carrier concentration to improve *σ* will reduce *S* and increase *κ_e_*; reducing the carrier concentration to enhance *S* will lower *σ*. In addition, bulk materials have a long lattice phonon mean free path, resulting in high *κ_l_*, which further reduces the *ZT* value. This coupling effect and high lattice thermal conductivity result in the low thermoelectric conversion efficiency of RTGs. Meanwhile, inefficient heat transfer and utilization between the radionuclide heat source and the thermoelectric material, caused by high thermal resistance at the material interface and low specific surface area, further reduce the overall energy conversion efficiency of RTGs.

### 2.2. Radio-Voltaic Batteries

Radio-voltaic batteries, including alpha-voltaic and beta-voltaic cells, directly convert the kinetic energy of charged particles emitted during radioactive decay into electrical energy using semiconductor transducers. This direct conversion approach enables miniaturization and seamless integration with integrated circuits, making such batteries well suited for microelectromechanical systems. The general conversion mechanism is based on the radio-voltaic effect: firstly, high-energy particles (alpha or beta particles) emitted by radionuclide decay bombard the semiconductor material, ionizing the lattice to generate a large number of electron-hole pairs (EHPs); secondly, the built-in electric field of the PN junction separates EHPs, driving electrons and holes to move to the n-type and p-type regions, respectively, forming an external current. Their fundamental mechanism is analogous to that of solar cells but relies on nuclear radiation as the energy source. The lifetime and efficiency of radio-voltaic batteries are determined by the characteristics of the semiconductor materials. Currently, radio-voltaic battery technology has achieved notable advances with over 20% conversion efficiency, which is several times higher than that of the RTG. However, most radio-voltaic batteries still exhibit low power output, delivering only nanowatt-level power for a 1 mCi isotope source. The underlying reason for this situation falls into three categories.

A main barrier is the severe self-absorption of decay particles. The radioisotope sources manufactured by current technologies, such as the powder metallurgic method, have a large thickness (μm to mm level), and short-range decay particles (e.g., alpha particles with a range of only a few micrometers in solids, low-energy beta particles) are easily absorbed by the source itself before escaping to the semiconductor, resulting in 70–90% decay energy loss. For bulk Ni-63 beta sources, the self-absorption loss of low-energy beta particles even exceeds 80%, which drastically reduces the radionuclide utilization rate.

The second bottleneck is the scale-length mismatch between radiation transport and the semiconductor transducer. The implantation depth of decay particles in semiconductors is fixed (related to particle energy), and the active region (depletion region) of bulk planar PN junctions is difficult to match with the particle implantation path. A large number of EHPs are generated in the non-active region and cannot be effectively collected, resulting in significant energy loss. For instance, in SiC-based batteries, the transport length of beta particles emitted from Y-90 (*E_max_* = 2.28 MeV) sources can be up to 2873 μm, whereas the maximum practical depletion width achievable through doping control is only about 2.6 μm. As a result, the majority of EHPs are generated outside the depletion region and subsequently lost to recombination. In contrast, alpha particles possess a much shorter range—approximately 20 μm for 5.3 MeV alpha particles in SiC—which aligns more favorably with the depletion width. However, the high linear energy transfer leads to severe radiation damage to the semiconductor lattice through displacement cascades; each incident alpha particle can generate hundreds of displacement events, causing rapid degradation of minority carrier lifetime and overall device performance. These limitations collectively hinder the technology from meeting medium- to high-power application requirements.

Finally, carrier collection efficiency is limited by the short range of the particles and the high recombination rate of EHPs. Planar bulk semiconductor PN junctions have a narrow depletion region and short carrier mean free path. EHPs generated by particle bombardment in the bulk region of the semiconductor are prone to recombination before reaching the depletion region, leading to a carrier collection efficiency of less than 30% for traditional planar silicon-based structures. In addition, bulk semiconductors have poor radiation resistance; long-term particle bombardment causes lattice distortion and point defects, which further increase carrier recombination and reduce collection efficiency.

In summary, the intrinsic limitations of bulk materials in thermoelectric and voltaic conversion mechanisms are the root causes of the low energy conversion efficiency, low radionuclide utilization rate, and poor stability of traditional radioisotope batteries. Nanomaterials and nanostructures break through these limitations by virtue of their unique structural and physical properties, which is the core logic of the application of nanotechnology in radioisotope batteries.

### 2.3. Radio-Photovoltaic Batteries

Radio-photovoltaic batteries, also referred to as radioluminescent or indirect conversion batteries, employ a two-step energy conversion process that physically decouples the radioactive source from radiation-sensitive electronic components. In this architecture, ionizing radiation (alpha, beta, or gamma) from the radioisotope first interacts with a luminescent material (scintillator/phosphor), exciting the material and triggering radiative de-excitation to emit photons. These photons are then collected by a photovoltaic (PV) cell, which converts light into electrical energy. This approach is particularly advantageous for alpha sources, as high-energy alpha particles are completely absorbed within the scintillator, preventing direct radiation damage to the PV cell. The overall efficiency of a radio-photovoltaic battery (denoted as *η*_total_) is the product of three sequential efficiencies: radiation-to-photon conversion efficiency (denoted as *η*_rad-to-light_), photon transport efficiency to the PV cell (denoted as *η*_optics_), and PV conversion efficiency (denoted as *η*_PV_), expressed as:

(1) $$\eta_{\mathrm{total}}\;={\;\eta }_{\mathrm{rad}\text{-}\mathrm{to}\text{-}\mathrm{light}}\cdot \eta_{\mathrm{optics}}\cdot \eta_{\mathrm{PV}}$$

The development of high-efficiency radio-photovoltaic batteries has been hindered by critical technical bottlenecks, predominantly related to scintillator performance. First, the radiation-to-photon conversion efficiency of conventional bulk phosphors (e.g., ZnS:Cu, rare-earth-doped oxides) is typically below 20%, as a significant fraction of decay energy is dissipated as heat via non-radiative processes. Second, many traditional scintillators exhibit self-absorption—emitted photons are reabsorbed by the material due to overlap between emission and absorption spectra, limiting light escape to the PV cell. Third, spectral mismatch between scintillator emission and PV cell response induces further conversion losses. Finally, prolonged irradiation (especially from intense alpha/beta sources) causes rapid degradation of luminescent properties in inorganic/organic scintillators, including color center formation and structural defects—this radioluminescence quenching reduces *η*_rad-to-light_ over time, limiting operational lifespan. For example, traditional ZnS-based phosphors show significant performance degradation under high-dose irradiation, rendering them unsuitable for long-term use. Additionally, efficient optical coupling between the scintillator and the PV cell introduces interfacial losses and fabrication complexity.

Nanotechnology offers a systematic pathway to overcome the key limitations of radio-photovoltaic batteries by improving each stage of the energy conversion chain. First, nanocomposite scintillators such as colloidal quantum dots and core–shell nanostructures exhibit high photoluminescence quantum yields (up to 90%), which significantly enhance the radiation-to-photon conversion efficiency (*η*_rad-to-light_) by suppressing non-radiative losses. Second, their size- and composition-dependent emission enables precise spectral tuning to match the external quantum efficiency of photovoltaic cells, thereby improving *η*_PV_ and reducing spectral mismatch losses. Third, engineered nanostructures can mitigate self-absorption through spatial separation of absorption and emission (e.g., core–shell designs), while highly transparent nanocomposite films reduce scattering and interfacial reflection, leading to improved photon transport efficiency (*η*_optics_). Furthermore, the incorporation of radionuclides at the nanoscale shortens energy transfer distances and promotes more uniform energy deposition. Importantly, nanomaterials also demonstrate enhanced radiation tolerance (e.g., quantum shells stable under X-ray doses exceeding 109 Gy), as surface passivation and structural confinement suppress defect formation and radioluminescence quenching under prolonged irradiation. Collectively, these advantages enable simultaneous improvements in efficiency and operational stability.

### 2.4. Other Radioisotope Batteries

Beyond the three primary categories, several specialized radioisotope battery concepts have been explored, each with distinct operating principles and niche applications. Direct charging batteries, one of the earliest concepts, operate by collecting charged particles (alpha/beta) from a radioisotope source on a separate electrode within a vacuum/low-pressure environment. Charge accumulation creates high potential differences (up to several kilovolts) but extremely low currents (nanoampere range), limiting practical use to specialized electrometer or ionization detector applications. Reciprocating cantilever (piezoelectric) nuclear batteries convert beta-source charge accumulation on a piezoelectric cantilever into mechanical oscillation—electrostatic force deflects the cantilever until contact with the source discharges it, enabling resonant oscillation and AC power generation. While innovative, this design suffers from low power density (microwatt range), mechanical fatigue of moving parts, and microscale integration complexity.

A more recent and promising alternative is the liquid-electrolyte/water-based nuclear battery, which leverages water radiolysis by ionizing radiation. Radiation decomposes water into reactive species (such as hydrated electrons e*_aq_^−^,* hydroxyl radicals ⋅OH, and hydrogen peroxide H_2_O_2_) that participate in electrochemical reactions at specialized electrodes, generating current. This approach circumvents solid-state semiconductor radiation damage, as the liquid medium is self-healing and replenishable. Studies using Sr-90/Y-90 beta sources and Pt/TiO_2_ Schottky junction electrodes have achieved power densities of tens of microwatts per square centimeter, but conversion efficiency remains below 1%, and radiolytic product management is critical for long-term safety. While these alternative technologies highlight diverse approaches to harnessing nuclear decay, their practical application remains limited.

Nanotechnology provides additional pathways to enhance the performance of emerging radioisotope battery systems, particularly in liquid-electrolyte configurations. Nanostructured electrodes (e.g., Pt/TiO_2_, or carbon-based nanomaterials) with high specific surface areas and tailored electronic structures can significantly improve the collection and separation efficiency of radiolytically generated charge carriers, thereby increasing effective current output. Surface functionalization and heterojunction design further promote selective redox reactions, suppressing recombination losses and enhancing overall conversion efficiency. In parallel, dispersing radioisotopes or scintillating nanoparticles at the nanoscale within the electrolyte shortens energy transfer distances and enables more uniform radiolysis, improving energy utilization efficiency. Nanomaterials also facilitate better control of reactive intermediates (e.g., e*_aq_*^−^, ·OH, H_2_O_2_) through catalytic or confinement effects, mitigating parasitic reactions and enhancing system stability. Moreover, the inherent structural tunability and radiation tolerance of nanomaterials contribute to prolonged operational lifetimes. Therefore, the current research focus remains on the three primary conversion mechanisms, where nanotechnology has demonstrated the most transformative impact on overcoming core limitations of low efficiency, poor stability, and scale-length mismatch.

## 3. Nanotechnologies in Radioisotope Batteries

### 3.1. Improving Energy Conversion Performance Through Nanomaterials

The innovation of nanomaterials is the core foundation for the performance improvement of radioisotope batteries. The performance of radioisotope batteries is directly determined by the physical and chemical properties of their core components, such as the radionuclide sources and energy conversion materials. From the perspective of energy conversion mechanisms, the efficiency of different types of radioisotope batteries is constrained by a series of scale-dependent processes, including phonon scattering in thermal transport, the energy deposition depth of radiation particles in materials (extinction/penetration depth), the carrier mean free path and recombination losses, as well as self-absorption within the radionuclide source. In conventional bulk materials, the characteristic dimensions are typically much larger than these intrinsic physical length scales, which often leads to excessively high thermal conductivity, insufficient carrier collection efficiency, and low utilization of radiation energy. By tuning material dimensions to be comparable to these characteristic scales, nanostructures enable synergistic optimization of thermal transport, charge carrier transport, and radiation energy deposition at the mechanistic level. In recent years, researchers have developed a variety of high-performance nanomaterials for different types of radioisotope batteries (RTGs, radio-voltaic batteries, radio-photovoltaic batteries, liquid electrolyte nuclear batteries, etc.), including thermoelectric nanomaterials, wide-bandgap semiconductor nanomaterials, nanocomposite luminescent materials, and nanoscale radionuclide source materials. Quantitative comparisons have been conducted on the performance parameters, applicable battery types, optimization mechanisms, and research outcomes of these nanomaterials, significantly advancing the development of isotope battery technology [1,9,19,26].

**Figure 3:**
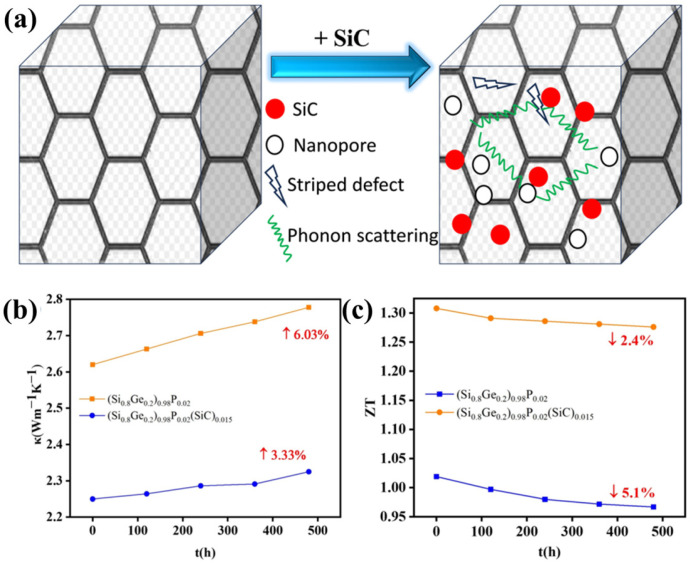
(**a**) Schematic diagram of nano defect formation and phonon scattering. Comparison of thermoelectric properties of undoped and SiC-doped samples after thermal aging: (**b**) Thermal conductivity, (**c**) ZT value [21].

In terms of thermoelectric nanomaterials for RTGs, silicon-germanium (SiGe) alloy nanomaterials are the most widely studied and applied due to their excellent high-temperature thermoelectric performance, good compatibility with nuclear radiation, and stable chemical properties. Recently, Zhao et al. prepared n-type nano-(Si_0.8_Ge_0.2_)_0.98_P_0.02_(SiC)_0.015_ alloy by high-energy ball milling combined with spark plasma sintering (SPS) (Figure 3) [21]. The study systematically clarified the influence of process parameters such as ball milling time, sintering temperature, and sintering pressure on the ZT value of the alloy, and achieved a ZT value of 1.308 at 1023 K, which is 30.8% higher than that of traditional bulk SiGe alloy (ZT = 1.0). This high-performance nanomaterial reduces lattice thermal conductivity through interfacial scattering effects of SiC nanoparticles, providing an efficient energy conversion material support for Pu-238-based RTGs used in deep space exploration. From a mechanistic perspective, nanoscale interfaces significantly enhance the scattering of mid- to long-wavelength phonons, thereby substantially reducing the lattice thermal conductivity without markedly degrading electrical conductivity, which in turn leads to an improved ZT value. Meanwhile, nanostructures can also introduce an energy filtering effect to a certain extent, further optimizing charge carrier transport. In addition to SiGe-based nanomaterials, bismuth telluride (Bi_2_Te_3_)-based heterostructure materials have also shown great application potential in medium-temperature RTGs [27,28]. L. D. Ivanova et al. constructed n-type Bi_2_Te_2.4_Se_0.6_-graphene heterostructure materials by melt spinning and crushing in a ball mill together with graphene plates, which achieved a ZT value of 1.3 at 420 K due to the enhanced phonon scattering effect of graphene and the improved carrier mobility of Bi_2_Te_3_ nanolayers [29]. This material with high ZT value can effectively improve the thermoelectric conversion efficiency of RTGs and actinide-based radioisotope batteries [27].

**Figure 4:**
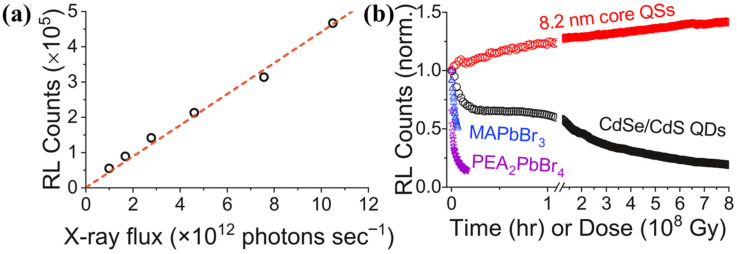
(**a**) X-ray flux-dependence of radioluminescence of core QS sample. (**b**) Time-dependent radioluminescence decays from QSs versus a conventional CdSe/CdS core/shell quantum dot sample and MAPbBr_3_ and PEA_2_PbBr_4_ halide perovskite crystals [37].

In a typical β-voltaic battery, semiconductors are a critical component. Consequently, many studies are focused on advanced semiconductor materials to achieve higher energy conversion efficiency [4,18]. Wide-bandgap semiconductor nanomaterials (such as diamond, SiC, and GaN) have become the core candidate materials for radioisotope battery energy conversion units due to their high breakdown field strength, excellent radiation resistance, and high temperature stability [30,31]. Han et al. reviewed the development status of diamond-based nuclear batteries and pointed out that diamond nanomaterials have the advantages of high carrier mobility (2000–2800 cm^2^/V·s), low dielectric constant, and good thermal conductivity, which can significantly improve the carrier collection efficiency and energy conversion efficiency of radioisotope batteries [32]. From the perspective of energy deposition and charge carrier transport mechanisms, the penetration depth of β particles in semiconductors is typically on the micrometer scale, whereas the carrier diffusion length and lifetime are limited, leading to substantial bulk recombination losses in conventional thick-junction structures. Nanoscale junction architectures (e.g., ultrathin p-n junctions or Schottky junctions) enable the device thickness to approach the carrier diffusion length, thereby significantly improving carrier collection probability. Meanwhile, reducing device thickness can also mitigate the detrimental impact of radiation-induced defects on overall device performance. The α-voltaic battery, combined with a Pu-238 radioactive source and a diamond Schottky device, achieved an energy conversion efficiency of 3.6%, which is the highest efficiency reported for α-voltaic batteries so far [33], and the γ-voltaic battery can obtain a maximum output power greater than 3 μW under the irradiation of a Cs-137 source [34]. Christopher Thomas et al. demonstrated that the tritium-based radioisotope β-voltaic batteries modified by 4H-SiC nanomaterials achieved a significant improvement in energy conversion efficiency, reaching 18.6%, which was attributed to the enhanced carrier collection capability of 4H-SiC nanomaterials [35]. This improvement fundamentally arises from the optimized electric field distribution and shortened carrier transport pathways in nanostructures, which reduce recombination probability and enhance charge collection efficiency. In addition to those nanomaterials, core–shell quantum dots (QDs), as a type of wide-bandgap semiconductor nanomaterial, also capture more attention with excellent radiation resistance [36,37,38]. The scintillation performance of colloidal quantum shells remained stable when irradiated by high-dose X-rays with a dose of >10^9^ Gy, which is much higher than that of traditional inorganic ceramic scintillators (scintillation performance degraded more than 80% under the same high-dose irradiation conditions), providing a reference for the radiation-resistant design of radioisotope batteries (Figure 4) [37].

Nanocomposite luminescent materials or nanoscale radionuclide source materials also provide nonnegligible help for the energy conversion performance [39,40,41]. Gui et al. investigated the influence of practical Ni-63 source thickness on self-absorption using Monte Carlo simulation and pointed out that nanoscale Ni-63 films (thickness < 0.5 μm) can significantly reduce the self-absorption loss of beta particles compared with bulk sources [41]. Its physical origin lies in the fact that when the β-particle range is on the same order of magnitude as the material thickness, an excessively thick source layer leads to complete energy dissipation within the source, whereas nanoscale thin films allow a larger fraction of particles to escape and participate in energy conversion, thereby increasing the externally usable flux. This reduction in self-absorption further leads to a several-fold improvement in the energy conversion efficiency of 4H-SiC microbatteries, providing a theoretical basis for the structural design of nanoscale Ni-63 sources. In line with this conclusion, Swastya Rahastama et al. established a self-absorption correction model for nanoscale Ni-63 beta sources [42]. Their findings indicated that the thinner the Ni-63 film, the higher the beta particle emission rate. Collectively, these two studies quantitatively verify the positive correlation between the thinness of Ni-63 sources and their performance, confirming that nanoscale Ni-63 films are the optimal choice for minimizing self-absorption loss and maximizing the utilization of beta particle energy, which also provides important quantitative and theoretical support for the optimization of high-performance β-voltaic battery structures.

### 3.2. Improving Energy Conversion Performance Through Nanostructure Regulation

Nanostructure regulation is an important means to optimize the performance of radioisotope batteries, as they can effectively improve the absorption efficiency of decay particles, the collection efficiency of carriers, and the stability of devices without changing the intrinsic properties of nanomaterials [17,33,43]. From a mechanistic perspective, this enhancement primarily arises from the regulation of key physical length scales by nanostructures, including the penetration depth of radiation particles, carrier diffusion length, and the spatial extent of the electric field. When the structural dimensions are commensurate with these characteristic scales, the spatial utilization efficiency of radiation energy deposition can be significantly improved, while the carrier transport pathways are shortened, thereby reducing recombination losses. By designing reasonable nanostructures (such as nanowire arrays, nanopillar arrays, and three-dimensional microstructures), the performance of radioisotope batteries can be significantly improved, which has become a research focus in the field of radioisotope batteries in recent years. A variety of nanostructure design strategies have been widely applied in different types of radioisotope batteries, achieving remarkable performance improvement effects [44].

In β-voltaic isotope batteries, nanostructure regulation has effectively solved the problem of low carrier collection efficiency of traditional planar structures, which is mainly due to the short range of β particles and the high recombination rate of carriers in planar structures [4]. Specifically, the energy deposition depth of β particles is typically on the micrometer scale, whereas conventional planar structures are relatively thick, resulting in substantial bulk recombination of carriers before they reach the junction region. Meanwhile, the electric field distribution in planar architectures is limited, making it difficult to effectively collect carriers generated far from the junction. He et al. designed a ^63^Ni-SiC-based 3D P^+^PNN^+^ multi-groove structure, which optimized the effective charge collection region of the semiconductor converter, resulting in a 45.64% carrier collection efficiency and a maximum output power density of 19.74 μW/cm^2^ under Ni-63 irradiation [44]. The reason is that the 3D multi-groove structure can effectively reduce the ridge spacing (0.8 μm) and width of the converter (1.2 μm), expand the specific surface area for isotope source loading, reduce the self-absorption effect of β particles by thinning the radioisotope source, and enable multidirectional interaction of β particles with the converter. Furthermore, the graded P/N epitaxial layer and low doping concentration (1 × 10^14^ cm^−3^) widen the depletion region (up to 7.58 μm) and extend the minority carrier diffusion length, thus reducing carrier recombination loss and improving the drift-diffusion and collection efficiency of electron–hole pairs. In addition, the coalescent energy transducer constructed by Li et al. achieved molecular-level coupling of radionuclides and energy transducer units, which can efficiently deposit the decay energy of α particles (about 5.27 MeV) emitted by Am-243 on the surrounding high-Z transducer units through inelastic collisions with extranuclear electrons of Tb atoms. This nanoscale coupled structure markedly shortens the energy transfer path of α particles, enabling high-energy particles to release their energy within an ultrashort distance. As a result, energy losses in non-active regions are reduced, and the conversion efficiency of radiation energy into usable photons or electrical energy is enhanced. This architecture enhanced the radiation-to-photon conversion efficiency of α decay energy to 3.43%, and the total power conversion efficiency of the integrated radio-photovoltaic micronuclear battery reached 0.889%, with a power per activity of 139 μW/Ci, providing a new technical route for the development of high-efficiency α-radioisotope micronuclear batteries and the resource utilization of actinides in nuclear waste [17].

### 3.3. Improving Energy Conversion Performance Through Interface Engineering

Interface engineering technology has played a crucial role in improving the stability of radioisotope batteries and reducing interface energy loss, which is one of the key factors affecting the long-term operation stability of devices. Interfacial micro- and nanostructures can optimize the energy deposition pathways of β particles, enabling a greater fraction of the radiation energy to be effectively absorbed within the semiconductor active region. Simultaneously, by regulating the electric field distribution and carrier transport channels, recombination losses are reduced, and the collection efficiency of EHPs is enhanced. Gao et al. designed a 3D interface simulation model with an inverted pyramid structure to forecast the performance of GaN-based β-voltaic nuclear batteries with PN junction 3D interface structures combined with practical machining processes [45]. The research used Geant4 to calculate the EHPs generation rate in GaN materials irradiated by Ni-63 and Pm-147 sources and employed COMSOL Multiphysics to simulate the EHPs transport phenomena in the battery and explore the influence of structural parameters on output performance. Simulation results indicate that the multidirectional surfaces of the inverted pyramidal structure enable multiple interactions between β particles and the semiconductor active layer, significantly increasing the generation rate of EHPs. Moreover, the inclined interfaces extend carrier drift paths under the electric field while reducing the average diffusion distance, effectively suppressing carrier recombination losses and thereby enhancing the overall device output performance. The results showed that with Ni-63 as the radioactive source, Na at 10^17^ cm^−3^, Nd at 10^14^ cm^−3^, a junction depth of 0.1 μm and inverted pyramid structures of 25, the battery achieved a short-circuit current density (Jsc) of 0.648 μA/cm^2^, an open-circuit voltage (V_oc_) of 2.3481 V and a maximum output power density (P_max_) of 1.2949 μW/cm^2^; when the radioactive source was replaced with Pm-147, the average J_sc_ increased to 56.865 μA/cm^2^ and the P_max_ rose to 94.975 μW/cm^2^, realizing a significant enhancement in output performance. The 3D inverted pyramid interface structure can nearly completely capture beta particles emitted by the radiation source, reduce directional energy loss, and significantly improve the collection efficiency of EHPs compared with planar interface structures. The increase in the number of inverted pyramid structures can boost the EHPs generation rate and thus the output performance of the transducer device. Furthermore, by appropriately tuning the thickness of the P region and the junction depth so that the device thickness approaches the carrier diffusion length, the separation and collection efficiency of EHPs can be further optimized, achieving a mechanistic improvement in carrier collection efficiency. The increase in P-region thickness will expand the junction depth, reduce the separation and collection efficiency of EHPs, leading to the linear decrease in J_sc_ and P_max_.

## 4. Application Progress of Radioisotope Batteries

With the support of nanotechnology, especially nanomaterial preparation and nanomanufacturing processes, the miniaturization and civilianization of radioisotope batteries have made significant progress in the past decades, breaking the traditional situation that radioisotope batteries are only used in aerospace and national defense special fields. Nanomaterials and nanomanufacturing processes, such as MEMS integrated manufacturing, electrochemical deposition, and CVD, have effectively solved the problem of balancing miniaturization and performance of traditional radioisotope batteries, promoting the application of radioisotope batteries from aerospace and national defense special fields to civilian fields such as medical treatment and IoT [3,7,30]. To improve clarity and enable systematic comparison, current technologies can be classified into three primary categories: direct conversion (alpha-voltaic, beta-voltaic), indirect conversion (radio-photovoltaic), and radiolysis-based systems (liquid-electrolyte batteries). Nanotechnology plays distinct roles across these categories, particularly in addressing efficiency, stability, and scaling limitations. Meanwhile, this section also presents a critical analysis of the application progress of nanomaterial-based radioisotope batteries in aerospace and civilian fields and compares the technical characteristics and application scenarios of typical devices.

### 4.1. Classification and Comparative Analysis of Radioisotope Batteries

Building on the categorization of current radioisotope battery technologies, representative device types and their performance characteristics are summarized as follows (Table 1). The table presents a systematic comparison of representative α/β-voltaic and radioisotope photovoltaic devices, highlighting their key performance parameters, structural features, and application suitability. This overview facilitates direct evaluation of technological trade-offs and practical capabilities across device types.

### 4.2. Aerospace Application: High Stability and Extreme Environment Adaptability

Aerospace is the most mature application field of radioisotope batteries, and nanomaterial-based RTGs and voltaic batteries have further improved the stability and energy conversion efficiency of aerospace nuclear batteries, adapting to the extreme deep space environment (ultra-low-temperature, high radiation, vacuum).

(1) Chang’E-3/4 lunar rovers: Adopted SiGe-based nanomaterial RTGs with a power of 0.1–2 W, which stably supplied power during the lunar night (ultra-low-temperature of −180 °C), verifying the reliability of nanomaterial-based RTGs in deep space exploration. The nano-SiGe alloy with a high ZT value effectively improved the thermoelectric conversion efficiency, ensuring the long-term operation of the rover under extreme temperature differences.

(2) SiC PIN nano-transducer α-voltaic batteries [46,47]: Gao et al. reported a significant advancement in alpha-voltaic cell technology through the development of a silicon carbide (SiC) PIN transducer. The proposed transducer features a sensitive region with an area of 1 cm^2^ and a width of 51.2 μm, achieving a charge collection efficiency of 95.6% at 0 V bias. By optimizing the unintentional doping concentration and crystal quality of the SiC epitaxial layer, a 2.4-fold enhancement in power conversion efficiency was achieved compared to previous studies. Electrical characterization, conducted using a He-ion accelerator as an equivalent alpha-radioisotope source, demonstrated a maximum power conversion efficiency of 2.10% and a maximum output power density of 406.66 nW cm^−2^. These results represent a substantial step toward the practical application of micronuclear batteries in microelectromechanical systems, micro aerial vehicles, and small satellites. The device exhibits robust operational stability across a wide temperature range, further supporting its potential for deployment in extreme environments [48].

Aerospace radioisotope batteries have high requirements for stability and radiation resistance, and nanomaterials (SiGe alloy, SiC, diamond) have become the core materials due to their excellent performance. However, the high preparation cost of aerospace-grade nanomaterials is acceptable for aerospace missions, but it is still a bottleneck for civilian applications.

### 4.3. Civilian Application: Miniaturization and Long-Term Maintenance-Free Operation

Driven by nanotechnology, radioisotope batteries have achieved significant breakthroughs in miniaturization and low power consumption, and typical civilian products (BV100 (Beijing Betavolt New Energy Technology Co., Ltd., Beijing, China), Zhulong No.1 (Wuxi Beta Pharmaceutical Technology Co., Ltd., Jiangyin, China)) have been developed, with broad application prospects in medical implants, IoT sensors, and deep-sea/polar exploration. A critical comparison of the two typical commercial civilian nuclear batteries is as follows:

(1) BV100 miniature nuclear battery (Beijing Betavolt): Adopts a nano-diamond semiconductor module and Ni-63 nanoscale source, with a volume of only 15 × 15 × 5 mm^3^ and a power of 100 μW at 3 V [49]. The core advantages are ultra-miniaturization and wide temperature adaptability (−60 °C to 120 °C), and it can operate continuously for 50 years without recharging or maintenance, suitable for cardiac pacemakers, IoT sensors in remote areas, and other low-power civilian scenarios. The nano-diamond module ensures high carrier collection efficiency, and the Ni-63 nanofilms reduce self-absorption loss, balancing miniaturization and performance.
(2) Zhulong No.1 C-14 nuclear battery: The world’s first C-14 nuclear battery based on SiC semiconductor nanomaterial design, leveraging the 5730-year half-life of C-14 to achieve an ultra-long theoretical lifespan. It has an ultra-high energy density of 2200 mWh/g (more than 10 times that of commercial lithium-ion batteries) and exceptional stability (performance decay < 5% over 50 years), with a working temperature range of −100 °C to 200 °C. Although the prototype’s output power is low (short-circuit current = 282 nA, Pmax = 433 nW), it has successfully driven an LED for nearly four months and a Bluetooth chip for signal transmission, verifying its application potential in implantable medical devices, deep-sea IoT sensors, and polar exploration equipment.

Civilian radioisotope batteries have achieved breakthroughs in miniaturization and long-term operation, but their output power is still low (μW/nW level), suitable only for low-power devices. The key challenges for civilian application are to improve the output power while maintaining miniaturization, and to reduce the preparation cost of nanomaterials and nanoscale radionuclide sources. In addition, the safety and standardization of civilian nuclear batteries need to be further improved (e.g., radionuclide leakage prevention).

## 5. Research Challenges

### 5.1. Stability Under Extreme Environments

Although nanotechnology has significantly improved the stability and radiation resistance of radioisotope batteries, the stability of nanomaterial-based radioisotope batteries under extreme environments (such as high temperature, low temperature, high radiation dose rate, and harsh chemical environments) is still one of the key challenges restricting their large-scale application. Radioisotope batteries used in deep space exploration, nuclear waste disposal, and deep-sea exploration need to work stably for a long time under extreme conditions, which puts forward high requirements for the stability of nanomaterials and nanostructures. The main problems are the performance degradation of nanomaterials under extreme temperatures, the structural damage under long-term high-dose radiation, and the corrosion of nanostructures under harsh chemical environments.

On the one hand, high-temperature and low-temperature environments will cause significant changes in the structure and performance of nanomaterials, mainly due to the thermal expansion and contraction of nanomaterials and the change in crystal structure. The nano-diamond semiconductor module adopted by Beta-volt can realize a wide temperature working range of −60 °C to 120 °C, but it still faces the problem of performance attenuation in ultra-low-temperature or ultra-high-temperature environments, which is mainly due to the change of diamond grain boundary structure and the increase in carrier recombination rate. On the other hand, long-term high-dose radiation will cause defects in nanomaterials and nanostructures, such as lattice distortion, interface separation, and point defects, which will lead to the attenuation of energy conversion efficiency and output stability of devices.

In addition, the corrosion of harsh chemical environments (such as acid-base, oxidizing, and salt spray environments) will also damage the nanostructure of radioisotope batteries and reduce the stability of devices. The nanoscale radionuclide source materials are easily corroded by chemical substances due to their high specific surface area and high chemical activity, leading to the leakage of radionuclides and the failure of devices. Therefore, improving the stability of nanomaterials and nanostructures under extreme environments is an urgent problem to be solved in the field of radioisotope batteries.

### 5.2. Performance Consistency from Nanomaterials to Macroscopic Integration

Another major challenge in the application of nanotechnology in radioisotope batteries is the performance consistency from nanomaterials to macroscopic integration, which is a key bottleneck restricting the industrialization of radioisotope batteries. At present, most of the research on nanomaterials for radioisotope batteries is carried out at the laboratory scale, and the prepared nanomaterials have excellent performance, but when these nanomaterials are integrated into macroscopic radioisotope battery devices, significant performance attenuation and inconsistency often occur, which restricts the large-scale production and application of high-performance radioisotope batteries.

The poor performance consistency is attributed to the following two reasons. First, the size and morphology of nanomaterials prepared by existing nanomanufacturing processes (such as high-energy ball milling, electrochemical deposition, and ALD) are difficult to achieve absolute uniformity, which leads to differences in the performance of individual nanomaterials. In addition, these differences are amplified during macroscopic integration, resulting in inconsistent performance of the entire device. For example, the particle size of n-type nano-(Si_0.8_Ge_0.2_)_0.98_P_0.02_(SiC)_0.015_ alloy prepared by high-energy ball milling is not uniform, which leads to differences in the thermoelectric performance of different regions of the macroscopic thermoelectric module, affecting the overall energy conversion efficiency of RTGs. Second, the interface between nanomaterials and substrate materials is difficult to achieve perfect bonding during macroscopic integration, resulting in interface defects and energy loss, which further reduces the performance of the device. For example, when silicon nanowire arrays are integrated into β-voltaic batteries, the interface between nanowires and the substrate often has defects such as gaps and impurities, which affect the collection efficiency of carriers.

In addition, the scalability of nanomanufacturing processes is a key factor affecting performance consistency. Most of the existing nanomanufacturing processes (such as CVD and ALD) are difficult to scale up, and the performance of nanomaterials prepared in large quantities is difficult to keep consistent with that of laboratory-scale samples. Therefore, developing scalable nanomanufacturing processes and improving the performance consistency of nanomaterials during macroscopic integration are important challenges to promote the industrialization of radioisotope batteries.

## 6. Conclusions and Outlook

### 6.1. Conclusions

In the past decades, nanotechnology has shown great application potential in the field of radioisotope batteries and has become a core driving force for the development and performance improvement of radioisotope battery technology. This review systematically summarizes the application progress of nanotechnology and nanomaterials in radioisotope batteries, covering the innovation of nanomaterial systems, the breakthrough of nanostructure regulation and interface engineering, and the progress of miniaturization and civilianization, which fully reflects the important role of nanotechnology in solving the technical bottlenecks of traditional radioisotope batteries.

The innovation of nanomaterial systems (including thermoelectric nanomaterials, wide-bandgap semiconductor nanomaterials, nanocomposite luminescent materials, and nanoscale radionuclide source materials) has fundamentally improved the energy conversion efficiency and stability of radioisotope batteries. For instance, the n-type nano-(Si_0.8_Ge_0.2_)_0.98_P_0.02_(SiC)_0.015_ alloy (ZT = 1.308 at 1023 K), Bi_2_Te_3_-based heterostructure materials, diamond nanomaterials, and other high-performance nanomaterials have provided efficient material support for different types of radioisotope batteries. The breakthrough of nanostructure regulation and interface engineering has effectively solved the problems of low carrier collection efficiency and poor stability of traditional radioisotope batteries, significantly enhancing device performance by optimizing radiation energy deposition pathways and carrier transport processes. The application of nanotechnology has also promoted the miniaturization and civilianization of radioisotope batteries, and the BV100 miniature nuclear battery and “Zhulong No.1” C-14 nuclear battery have laid a solid foundation for the civilian application of radioisotope batteries.

However, it should be noted that the application of nanotechnology in radioisotope batteries still faces many challenges, including the stability under extreme environments (high temperature, low temperature, high radiation, and harsh chemicals), the performance consistency from nanomaterials to macroscopic integration, the high cost of nanomaterials and manufacturing processes, and the safety and standardization issues. These challenges restrict the large-scale application and industrialization development of nanomaterial-based radioisotope batteries and need to be solved through in-depth research in the future.

### 6.2. Outlook

Based on the current research progress and existing challenges, the future development direction of nanotechnology in the field of radioisotope batteries will focus on solving the existing technical bottlenecks, promoting the performance improvement and industrialization of radioisotope batteries, and expanding their application fields. The main development directions are as follows:

Firstly, the development of new high-performance nanomaterial systems is desired. On the one hand, researchers should continue to optimize the structure and performance of existing nanomaterials (such as SiGe-based nanomaterials, diamond nanomaterials, and SiC nanomaterials) through precise regulation of size, morphology, and interface properties, further improving their energy conversion efficiency and radiation resistance. On the other hand, researchers should develop new types of nanomaterials, such as two-dimensional nanomaterials and perovskite nanomaterials, exploring their application potential in radioisotope batteries. For example, MXenes nanomaterials have high electrical conductivity and thermal conductivity, which are expected to be used in high-efficiency thermoelectric conversion layers. In addition, the development of nanocomposite materials with multi-functional integration will also become a research focus, meeting the needs of multi-scenario applications of radioisotope batteries.

Secondly, the innovation of nanostructure design and interface engineering technology should not be ignored. It is necessary to develop more efficient nanostructure design strategies, such as three-dimensional ordered nanostructures and hierarchical nanostructures, further improving the absorption efficiency of decay particles and the collection efficiency of carriers. At the same time, researchers should explore new interface modification technologies, such as atomic layer etching and in situ interface growth, to achieve perfect bonding between nanomaterials and substrate materials, reducing interface defects and energy loss. In addition, the combination of nanostructure regulation and interface engineering with artificial intelligence (AI) technology will also become a new development trend, realizing the intelligent design and optimization of nanostructures and interfaces.

Thirdly, the development of scalable nanomanufacturing processes is essential. Researchers should focus on solving the problem of poor scalability of existing nanomanufacturing processes, developing low-cost, large-scale nanomanufacturing technologies, and improving the performance consistency of nanomaterials during large-scale production and macroscopic integration. The combination of nanomanufacturing processes with MEMS and microfabrication technologies will also promote the miniaturization and integration of radioisotope batteries, expanding their application in microelectronic devices and implantable medical equipment.

Fourthly, the improvement of the stability and safety of nanomaterial-based radioisotope batteries is indispensable. Researchers should carry out in-depth research on the performance attenuation mechanism of nanomaterials under extreme environments and long-term radiation and develop effective stability improvement technologies. At the same time, researchers should strengthen research on the safety of nanomaterial-based radioisotope batteries, developing reliable radionuclide leakage prevention technologies and safety testing methods, ensuring their safe application in civilian fields. In addition, the establishment of a standardized system for nanomaterial-based radioisotope batteries will also promote the healthy development of the industry, which requires close cooperation between governments, research institutions, and enterprises.

In conclusion, nanotechnology has brought unprecedented opportunities for the development of radioisotope battery technology and has made remarkable progress in the past decades. Although there are still many challenges to be solved, with the in-depth development of nanoscience and technology, the continuous innovation of nanomaterials and nanomanufacturing processes, nanomaterial-based radioisotope batteries will surely achieve greater breakthroughs in performance improvement, industrialization, and application expansion. They will become a core energy solution for extreme environments and long-term energy supply scenarios and make important contributions to the development of aerospace, medical care, new energy, and other fields.

## Figures and Tables

**Table 1:** Comparison of performance between typical research results and commercial products for isotope batteries.

Type	Material System	Performance Metrics	Advantages	Limitations
alpha-voltaic	SiC PIN device	2.10%, 406.66 nW·cm^−2^	High stability, radiation resistance	Low efficiency
beta-voltaic	Ni-63 diamond	~100 μW	Long lifetime, miniaturization	Efficiency unclear
	C-14 SiC	433 nW, 282 nA	Ultra-long lifetime, high energy density	Extremely low output current
radiolysis	Sr-90/Pt-TiO_2_	<1%, μW·cm^−2^	Self-healing system	Low efficiency, system complexity
radio-photovoltaic	^3^H/ZnS:Cu + Si	~0.1–1%, μW·cm^−2^	Good radiation shielding, flexible design	Optical losses, low light intensity

## Data Availability

No new data were created or analyzed in this study.

## Abbreviations

The following abbreviations are used in this manuscript:

RTGs	Radioisotope thermoelectric generators
CVD	Chemical vapor deposition
ALD	Atomic layer deposition
QDs	Quantum dots
PLQY	Photoluminescence quantum yield
HCNT	Hollow carbon nanotube
CNTs	Carbon nanotubes
IoT	Internet of Things
